# Identification of Anti-Influenza A Compounds Inhibiting the Viral Non-Structural Protein 1 (NS1) Using a Type I Interferon-Driven Screening Strategy

**DOI:** 10.3390/ijms241310495

**Published:** 2023-06-22

**Authors:** Giulia Marsili, Chiara Acchioni, Anna Lisa Remoli, Donatella Amatore, Rossella Sgarbanti, Marta De Angelis, Roberto Orsatti, Marta Acchioni, Andrea Astolfi, Nunzio Iraci, Simona Puzelli, Marzia Facchini, Edvige Perrotti, Violetta Cecchetti, Stefano Sabatini, Fabiana Superti, Mariangela Agamennone, Maria Letizia Barreca, John Hiscott, Lucia Nencioni, Marco Sgarbanti

**Affiliations:** 1 Department of Infectious Diseases, Istituto Superiore di Sanità, Viale Regina Elena 299, 00161 Rome, Italy; giulia.marsili@iss.it (G.M.); chiara.acchioni@iss.it (C.A.); aremoli@gmail.com (A.L.R.); roberto.orsatti@iss.it (R.O.); marta.acchioni@iss.it (M.A.); simona.puzelli@iss.it (S.P.); marzia.facchini@iss.it (M.F.); edvige.perrotti@iss.it (E.P.); 2 Department of Public Health and Infectious Diseases, Laboratory Affiliated to Istituto Pasteur Italia-Fondazione Cenci Bolognetti, Sapienza University, 00185 Rome, Italy; amatore.donatella@gmail.com (D.A.); marta.deangelis@uniroma1.it (M.D.A.); rossella.sgarbanti@gmail.com (R.S.); lucia.nencioni@uniroma1.it (L.N.); 3 Laboratory of Virology, Department of Molecular Medicine, Sapienza University of Rome, 00185 Rome, Italy; 4 Department of Pharmaceutical Sciences, Università degli Studi di Perugia, Via del Liceo 1, 06123 Perugia, Italy; andrea.astolfi@unipg.it (A.A.); violetta.cecchetti@unipg.it (V.C.); stefano.sabatini@unipg.it (S.S.); maria.barreca@unipg.it (M.L.B.); 5 Department of Chemical, Biological, Pharmaceutical and Environmental Sciences, University of Messina, Viale Ferdinando Stagno d’Alcontres 31, 98166 Messina, Italy; nunzio.iraci@unime.it; 6 National Centre for Innovative Technologies in Public Health, Istituto Superiore di Sanità, Viale Regina Elena 299, 00161 Rome, Italy; fabiana.superti@iss.it; 7 Department of Pharmacy, University “G. d’Annunzio” of Chieti-Pescara, Via dei Vestini 31, 66100 Chieti, Italy; m.agamennone@unich.it; 8 Istituto Pasteur Italia, Fondazione Cenci Bolognetti, Viale Regina Elena 291, 00161 Rome, Italy; john.hiscott@istitutopasteur.it

**Keywords:** influenza A viruses, NS1, type I IFN, small molecule screening, luciferase reporter assay, diverse compound library, pharmacophore modeling

## Abstract

There is an urgent need to identify efficient antiviral compounds to combat existing and emerging RNA virus infections, particularly those related to seasonal and pandemic influenza outbreaks. While inhibitors of the influenza viral integral membrane proton channel protein (M2), neuraminidase (NA), and cap-dependent endonuclease are available, circulating influenza viruses acquire resistance over time. Thus, the need for the development of additional anti-influenza drugs with novel mechanisms of action exists. In the present study, a cell-based screening assay and a small molecule library were used to screen for activities that antagonized influenza A non-structural protein 1 (NS1), a highly conserved, multifunctional accessory protein that inhibits the type I interferon response against influenza. Two potential anti-influenza agents, compounds **157** and **164**, were identified with anti-NS1 activity, resulting in the reduction of A/PR/8/34(H1N1) influenza A virus replication and the restoration of IFN-β expression in human lung epithelial A549 cells. A 3D pharmacophore modeling study of the active compounds provided a glimpse of the structural motifs that may contribute to anti-influenza virus activity. This screening approach is amenable to a broader analysis of small molecule compounds to inhibit other viral targets.

## 1. Introduction

Influenza viruses are important human pathogens infecting up to 500 million people annually, resulting in an estimated 250,000–500,000 deaths worldwide [1], with ~80% of those deaths occurring in the elderly population. The 2009 H1N1 influenza pandemic caused more than 18,000 deaths [2] and illustrated the rapidity with which the spread of influenza could become global. Before the outbreak of COVID-19, influenza viruses were thought to be the source of the next deadly pandemic. Moreover, avian influenza viruses also pose a potential threat to human health [3], as demonstrated by the outbreak of the A(H5N1) virus in 1997 [4] and the human infections with 2013 influenza A(H7N9) in China [5]. Influenza viruses belong to the *Orthomyxoviridae* family and possess a negative-strand segmented RNA genome (7 or 8 single-stranded RNA segments encoding 12 proteins). They are divided into four distinct types, A, B, C, and D [6], among which types A and B are globally distributed pathogens causing respiratory disease in humans [7]. The influenza virus can trigger pulmonary inflammation and exacerbates chronic lung diseases, with infiltration of inflammatory cells and increased airway hyperresponsiveness. Bronchial epithelial cells, the primary target and the main host cells for the influenza virus, play an important role in the pathogenesis of this infection [1].

To date, three classes of anti-influenza drugs are available: inhibitors of the integral membrane proton channel protein M2 (adamantanes), inhibitors of the neuraminidase (NA) protein (oseltamivir, zanamivir, peramivir and laninamivir), and inhibitors of RNA-dependent RNA polymerase such as polymerase basic protein 1 (PB1) inhibitor (favipiravir) and polymerase acidic protein (PA) inhibitor (baloxavir marboxil). Influenza A(H1N1)pdm09 and A(H3N2) circulating viruses are resistant to the adamantane activity, and H1N1 viruses retained fitness after acquiring resistance to oseltamivir [8]. Moreover, reduced susceptibility to baloxavir marboxil has been reported for influenza A(H1N1)pdm09 and influenza A(H3N2) viruses carrying an I38T mutation in the polymerase acidic protein [9]. Finally, the acquisition of favipiravir resistance in the pandemic H1N1 influenza A virus has been demonstrated in a laboratory setting [10], and the transmission of favipiravir-resistant viruses has been observed in vivo [11], thus highlighting the urgent need to develop anti-influenza drugs with unique mechanisms of action, potentially as a first line of defense against a serious or pandemic outbreak.

Influenza A non-structural protein 1 (NS1), encoded by segment eight genomic vRNA, exerts multiple accessory functions during viral infection; the principal among these is the inhibition of host antiviral innate response [12]. NS1 is highly expressed in infected cells with a predominant nuclear localization and a significant cytoplasmic presence later in infection [13,14]. NS1 possesses a strain-dependent length of 230–237 aa and two different domains [15]. The NH2-terminal RNA-binding domain (residues 1-73) binds with low affinity to several RNA species in a sequence-independent manner [16,17,18,19]. The effector domain (ED) at the COOH-terminus (residues 74-230/237) mainly mediates interactions with host cell proteins [15]. NS1 antagonizes both interferon type I (IFN-I) production and the antiviral effect of IFN-stimulated gene (ISG) products at both the transcriptional [20,21,22] and post-transcriptional level [23,24]. Pre-transcriptional inhibition of IFN-I induction is related to the block of virus-mediated activation of IRF-3, IRF-7, and NF-κB cellular transcription factors through the direct binding to the RNA sensor RIG-I [25,26] and also by inhibiting RIG-I ubiquitination [25,27,28,29]. NS1 can also block the function of two ISGs whose products are localized in the cytoplasm and possess antiviral activities, i.e., the dsRNA-dependent serine/threonine protein kinase R (PKR) and the 2′-5′-oligoadenylate synthase (OAS), by directly binding PKR [30] and indirectly preventing viral RNAs from triggering OAS activation [30,31]. Post-transcriptional inhibition of the IFN-I response is obtained via interaction of the NS1 ED with both the 30-kDa subunit of the cleavage and polyadenylation specificity factor (CPSF30), a nuclear protein required for 3′ end processing of cellular pre-mRNAs [32,33], and the poly(A)-binding protein II (PABP II) involved in the nuclear export of fully processed mRNAs [34]. NS1 also acts by interfering with NXF1/TAP and several proteins of the nuclear export machinery [35]. The resulting inhibition of nucleo- to cytoplasmic transport of all cellular poly(A)-containing mRNAs also indirectly blocks the translation of IFN-I mRNA [32].

In the present study, an IFN-β promoter luciferase cell-based screening assay was used to identify inhibitors of influenza NS1. From a diverse library of small molecules, two potential anti-influenza agents were identified that antagonized A/PR/8/34(H1N1) replication in human lung epithelial A549 cells, resulting in the restoration of IFN-β and ISGs expression in virus-infected cells.

## 2. Results

### 2.1. Cell-Based Assay to Identify Potential Anti-Influenza A NS1 Compounds 

**Figure 1:**
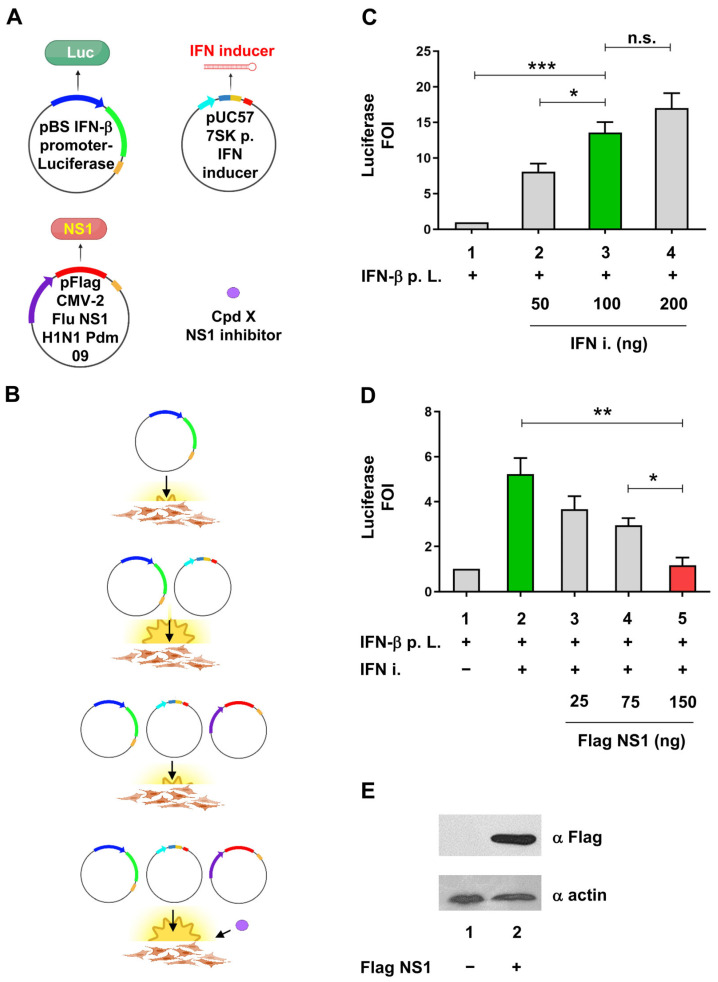
Cell-based assay to evaluate the anti-IFN-I activity of NS1. (**A**) Schematic representation of plasmids, small molecules, and experimental points used for the cellular luciferase assay. The pBS IFN-β promoter-Luciferase reporter construct, the pUC57 nt shRNA (IFN-inducer), and the pFlag CMV2 NS1 (H1N1 Pdm 09) are shown. RNA Pol II promoter regions of IFN-β and the CMV virus promoters are shown as tick blue and violet arrows, respectively. RNA Pol III 7SK promoter is shown as a tick light blue arrow. Coding regions for luciferase and NS1 are shown as tick green and red rectangles, respectively. The forward and reverse sequences belonging to the nt shRNA IFN inducer are shown as petroleum and magenta rectangles, respectively. Compound x, able to act as an NS1 inhibitor, is shown as a purple-filled circle. RNA Pol II PolyA sequences and RNA Pol III terminator region are shown as mustard and red rectangles, respectively. The nt shRNA IFN inducer is shown as a 35 bp long red hairpin structure; pBS = pBluescript; 7SK p. = 7SK promoter; H1N1Pdm 09 = A(H1N1)pdm09 influenza virus; compound (Cpd). (**B**) Schematic representation of the expected outcome of transfection with different plasmid combinations and effective compound Cpd x treatment. The basal and induced luminescence signals of the IFN-β promoter luciferase construct, transfected in IFN-I competent cells alone or together with the nt shRNA (IFN inducer) expressing vector, are shown. The inhibition of luciferase signal after NS1 expressing vector co-transfection and the restoration of luciferase expression by the pretreatment with an x compound are also shown schematically. (**A**,**B**) were created with BioRender.com (accessed on 5 June 2023). (**C**) Evaluation of different IFN-inducer construct amounts to obtain an optimal IFN-β stimulation suitable for the assay. HEK 293 cells were transiently transfected with 25 ng of IFN-β promoter luciferase reporter construct alone (IFN-β p. L.) or together with increasing amounts of shRNA plasmid expressing the IFN-inducer (IFN-i). (**D**) Evaluation of the optimal NS1 expressing construct to be used in the assay. HEK 293 cells were transiently transfected with increasing amounts of pFLAG CMV2 expression A(H1N1) pdm09 NS1, together or not with the same amounts of IFN-β p. L. and IFN-I constructs used in (**A**). The green bars in both panels C and D indicate the optimal induction of the luciferase signal by the IFN-inducer (IFN-i) expression, while the red bars in panel D indicate the optimal inhibition of luciferase signal by the co-transfection of NS1 expression vector. IFN-β p. L. = IFN-β promoter Luciferase. FOI = Fold of Induction. (**E**) Expression of NS1 after transfection by Western blotting using anti-FLAG (NS1) and anti-actin (as a loading control) Antibodies. HEK 293 cells were transiently transfected with 150 ng of pFLAG CMV2 NS1 and WCE subjected to Western blotting with αFLAG monoclonal Ab, using actin as loading control, detected with αActin polyclonal Ab. Results shown in the bar graph are expressed as luciferase FOI with respect to cells transfected with the empty vector. Mean and standard deviation are shown. Statistical analysis was performed using the “two tailed unpaired *t* test” ((**A**), left panel). * *p* < 0.05; ** *p* < 0.01; *** *p* < 0.001; n.s. not significant.

To test the anti-NS1 activity of small molecules, the IFN-β gene promoter driving the luciferase reporter gene [36] was co-expressed together with a plasmid encoding for a 35 bp not-targeting (nt) short hairpin (sh) RNA [37], named herein as “IFN inducer”, and an expression vector for A(H1N1)pdm09 NS1. As depicted in Figure 1A,B, transient co-transfection of the IFN-inducer plasmid into HEK 293 cells will result in an increase of the luciferase signal generated as a consequence of the IFN-β promoter stimulation. The co-transfection of the NS1-expressing plasmid will decrease luminescence due to the inhibition of the IFN signal transduction pathway. Finally, the addition of a potential NS1 inhibitor will restore the inducibility of the IFN-β promoter by the IFN-inducer.

Expression vector for NS1 protein belonging to A(H1N1)pdm09 pandemic influenza A subtype was evaluated in the cell-based assay for its ability to inhibit the stimulation of the IFN-β promoter by the IFN-inducer construct. To obtain a consistent stimulation of the IFN-β promoter and a concomitant inhibition by NS1, different amounts of IFN-inducer and NS1-expressing plasmids were evaluated in the experimental setup. The 100 ng amount of the IFN inducer plasmid (Figure 1C), coupled with 150 ng of NS1 plasmid (Figure 1D), were chosen based on their statistically significant activation and inhibition, respectively; the activation of IFN-β promoter by the IFN inducer RNA was significantly reduced (77.7%) by NS1 expression, the latter detected by immunoblotting (Figure 1D,E).

### 2.2. Identification of Compounds Inhibiting NS1 activity in the Cell-Based Assay

**Figure 2:**
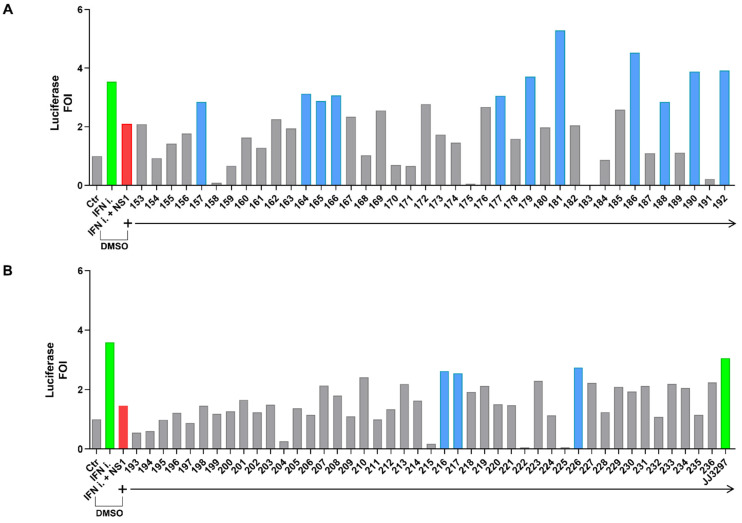
(**A**,**B**) Screening of a diverse library of compounds in the IFN-β luciferase system (treatment with compounds from 153 to 192 is shown in panel (**A**), while treatment with compounds from 193 to 236 is shown in panel (**B**)). (**A**) HEK 293 cells were transiently transfected with the IFN-β promoter luciferase reporter construct alone, together with the IFN-inducer shRNA plasmid alone, or together with the pFLAG CMV2 A(H1N1)pdm09 NS1 construct. Cpds 153 to 192 of the library were added to cells at a final concentration of 50 μM in fresh medium 6 h post-transfection, and cells were harvested 42 h later (48 h after transfection) and subjected to luciferase assay. (**B**). HEK 293 cells were transiently transfected as in A, and cpds 193 to 236 of the library, were added to cells as in (**A**). The reference cpd **JJ3297** was used at 5 μM. The empty vector pFLAG CMV2 was used to normalize the amount of transfected DNA in each experimental point. Results shown in the bar graph are expressed as luciferase fold of induction (FOI) with respect to cells transfected with the empty vector. Green and red bars indicate the upregulation and downregulation of luciferase activity, respectively. Blue bars underline >50% restoration obtained with the effective compounds. All experimental points were transfected with the IFN-β promoter reporter construct. The green bars in both panels indicate the induction of the luciferase signal by the IFN-inducer (IFN-i) expression (in panel (**B**), the far-right green bar also indicates the treatment with positive control **JJ3297**). DMSO (Dimethyl sulfoxide) presence is indicated in both panels.

Compounds belonging to a diverse library of 84 small molecules [38] were tested in the experimental system at a concentration of 50 μM using a luciferase glow detection assay (Figure 2A,B). Several compounds—**157**, **164**, **165**, **166**, **177**, **179**, **181**, **186**, **188**, **190**, **192**, **216**, **217,** and **226**—increased IFN-β promoter stimulation by at least 50%. The specificity of the IFN-β promoter assay was also confirmed with reference compound **JJ3297**, a known NS1 inhibitor (right side of Figure 2 panel B) [39].

**Figure 3:**
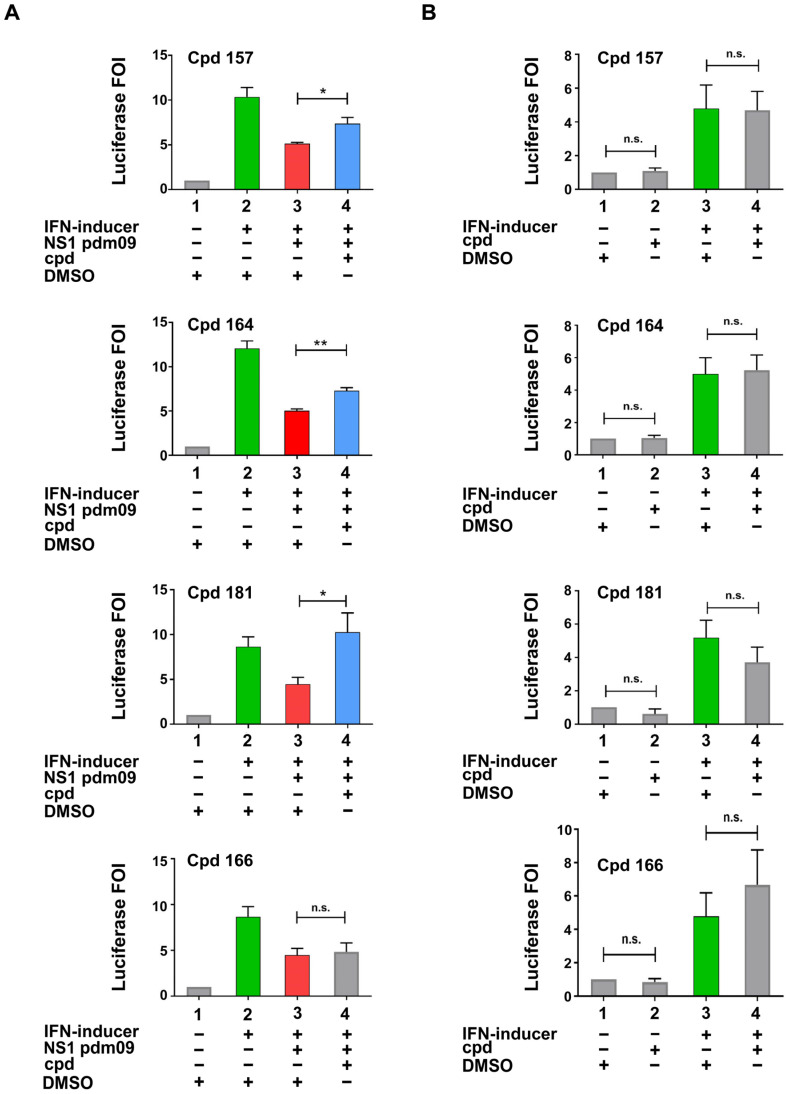
Characterization of inhibitory compounds (**A**,**B**). (**A**) Confirmation experiments of compounds identified as effective in the screening. (**B**) Specificity test of the same compounds analysed in A in the absence of NS1 expressing plasmid; compound = Cpd. DMSO (Dimethyl sulfoxide) presence is indicated in both panels. H1N1Pdm 09 = A(H1N1)pdm09 influenza virus. (**A**,**B**) HEK 293 cells were transiently transfected with the IFN-β promoter luciferase reporter construct alone, together with the IFN-inducer nt shRNA plasmid alone, or together with the pFLAG CMV2 A(H1N1)pdm09 NS1 construct. Compounds testing positive by luciferase analysis were added to cells at a final concentration of 50 μM in fresh medium 6 h post-transfection, and cells were harvested 42 h later (48 h after transfection) and subjected to luciferase assay. Results shown in the bar graph are expressed as luciferase fold of induction (FOI) relative to cells transfected with the empty vector. Green and red bars indicate the upregulation and downregulation of luciferase activity, respectively. Blue bars underline restoration obtained with the effective compounds. Mean and standard deviation are shown. Statistical analysis was performed using the “two tailed paired *t* test”. * *p* < 0.05, ** *p* < 0.01, n.s. not significant.

Compounds that tested positive in the screening were re-evaluated with a more sensitive flash luciferase detection assay in the same IFN-β promoter stimulation assay. As shown in Figure 3A, compounds **157**, **164,** and **181** demonstrated a significant restoration of the luciferase signal (compound 166 was shown as an example of an unconfirmed positive hit). To rule out potential non-specific activities within the assay, compounds were tested in the absence of NS1 while in the presence or absence of the IFN inducer (Figure 3B); these results showed no statistically significant difference compared to controls.

### 2.3. Compounds 157 and 164 Inhibit Influenza A/PR/8/34(H1N1) Virus Replication and Restore IFN-β and IFN-Stimulated Gene (ISGs) Expression in Infected Cells

**Figure 4:**
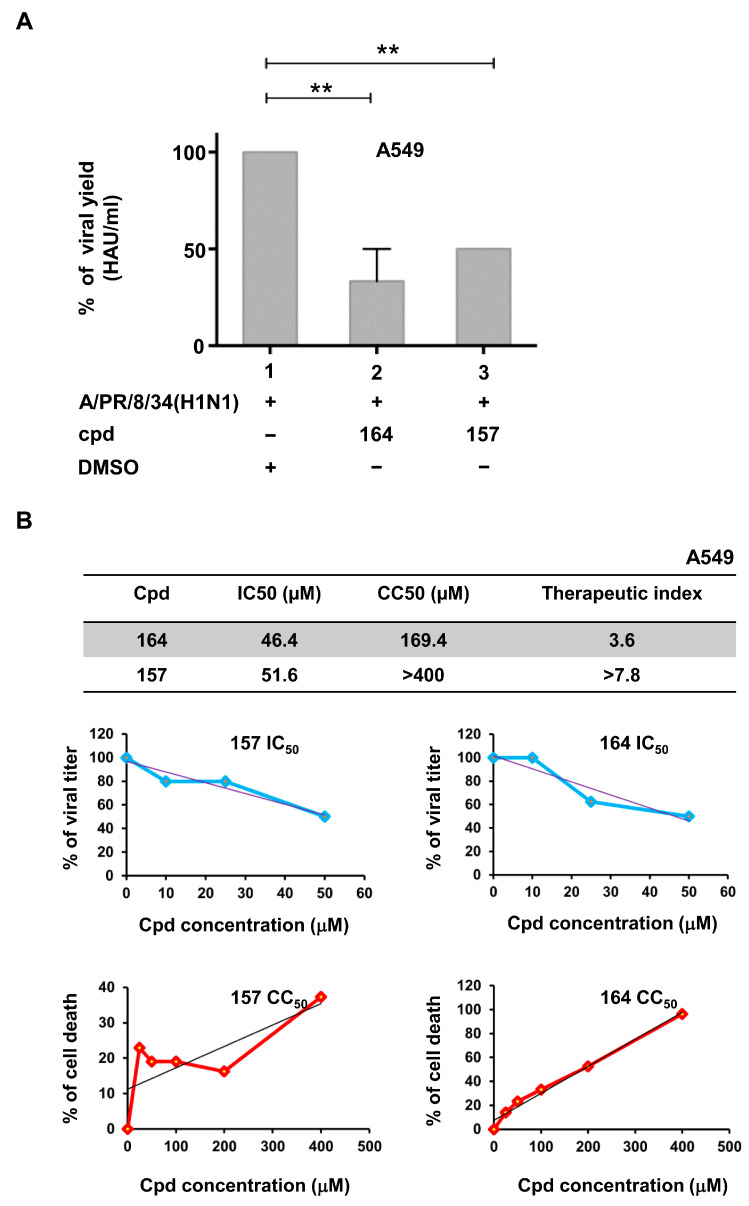
Compounds **157** and **164** inhibit influenza virus replication. (**A**) Compounds **157** and **164** inhibit influenza virus replication. A549 cells were seeded at a density of 2 × 10^5^/mL in 24 multiwell plates for 24 h. Then, cells were treated with cpds **164** and **157** for 3 h and infected with influenza virus A/PR/8/34(H1N1) at an MOI of 0.1. After 24 h of infection, viral titer was evaluated in the supernatant of infected cells through hemagglutination assay. Results shown in the bar graph are expressed as % viral yield (HAU/mL). Means ± standard deviations from three separate experiments are shown. Statistical analysis was performed using the “one-way analysis of variance” (ANOVA), followed by the Newman–Keuls post hoc test. ** *p* < 0.01 vs. untreated cells. Cpd = compound. DMSO (Dimethyl sulfoxide) presence is indicated. (**B**) IC_50_, CC_50_, and therapeutic index of tested compounds are indicated in the A549 cellular system. Dose–response curves for both IC_50_ and CC_50_ are also shown in the upper and lower graphs of panel (**B**).

To determine if compounds testing positive in the cell-based assay were able to inhibit influenza A virus replication, the human lung epithelial A549 cells were treated for 3 h with compounds **157**, **164**, and **181** and then subjected to infection with 0.1 MOI of influenza virus A/PR/8/34(H1N1). Only compounds **157** and **164** inhibited viral replication (50% and 60% reduction, respectively), with IC_50_ values of 51.6 μM and 46.4 μM, respectively (Figure 4A,B). The cytotoxic concentration 50 (CC_50_) for **157** and **164** was calculated as >400 μM for **157** and >150 μM for **164**. Thus, the therapeutic index of **157** and **164**, defined as the ratio between CC_50_/IC_50_ values, was >7.8 and 3.6 in A549 cells, respectively (Figure 4B).

**Figure 5:**
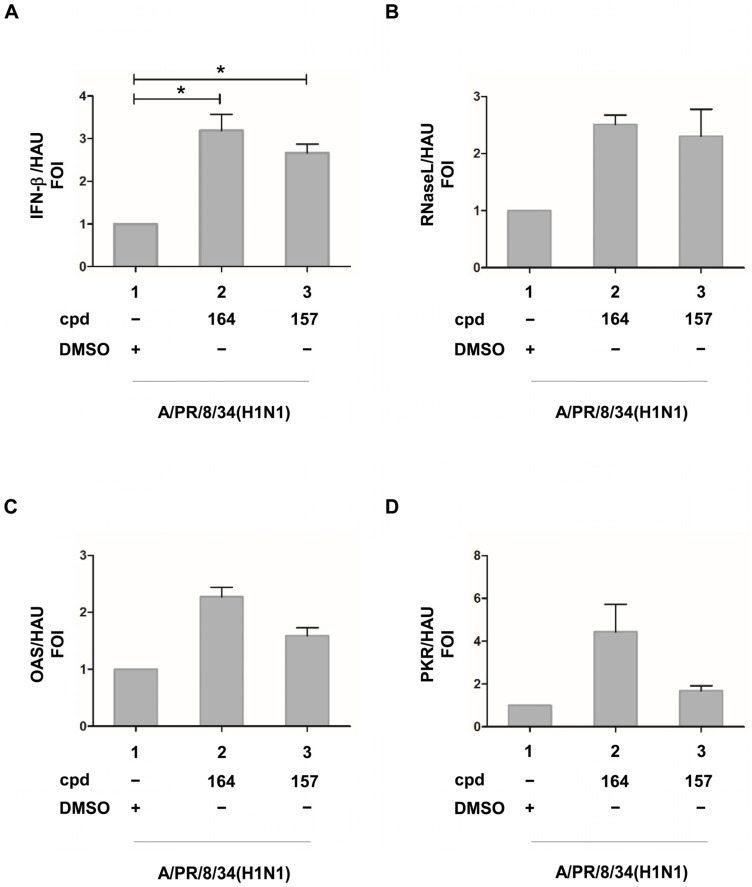
(**A**) IFN-β, (**B**) RNase L, (**C**) OAS, and (**D**) PKR ISGs expression in the presence of compounds **164** and **157** in influenza A/PR/8/34(H1N1)-infected cells upon normalization for HAU (hemagglutinating unit). A549 cells were seeded at a density of 2 × 10^5^/mL in 24 multiwell plates for 24 h. Then, cells were treated with compounds **164** and **157** for 3h and infected with influenza virus A/PR/8/34(H1N1) at an MOI of 0.1. At 24 h post-infection, total RNA was extracted and subjected to quantitative RT PCR to detect the accumulation of IFN-β (**A**), RNase L (**B**), OAS (**C**), and PKR (**D**) mRNA. Results are expressed as FOI (fold of induction) upon normalization with GAPDH mRNA and also with HAU (hemagglutination units). Means ± standard deviations from three separate experiments are shown (**A**,**B**). DMSO (Dimethyl sulfoxide) presence is indicated in all panels. Statistical analysis was performed using the “two tailed paired t test” ((**A**), left panel). * *p* < 0.05.

To determine if inhibition of viral replication by the two bioactive compounds was associated with an increase in IFN-β and IFN-I stimulated genes, mRNA accumulation in infected A549 cells was evaluated by qRT-PCR after normalization for virus replication with HAU. A significant 3.2- and 2.7-fold increase in IFN-β mRNA accumulation was observed with **164** and **157**, respectively (Figure 5A). A trend of increased ISGs expression was also detected in **157** and **164** treated cells, with a 2.5- and 2.3-fold increase for RNase L, 2.3 and 1.7 for 2′5′ OAS, and 4.5 and 1.7 for PKR, respectively (Figure 5B–D).

Finally, the key physicochemical and pharmacokinetic properties, as well as the ADME (adsorption, distribution, metabolism, and excretion) parameters of **157** and **164,** were predicted through the free web tool SwissADME (http://www.swissadme.ch (accessed on 3 May 2023) [40]. The outcomes (Figure S1) suggested a drug-like profile and no alerts (e.g., the presence of Pan Assay Interfering compounds (PAINS) structures) for both small molecules.

## 3. Discussion

NS1 is a multifunctional regulatory protein that readily inhibits the antiviral response by counteracting the production of type I IFN, often in a strain-specific manner [41], and/or inhibiting the activity of ISGs. Previous studies demonstrated that deletion of NS1 from influenza A/H1N1 generated a recombinant virus with lower pathogenicity in rhesus macaques and with the capacity to protect animals from a lethal challenge with wild-type influenza [42]. Thus, NS1 represents an ideal therapeutic target for small molecule inhibition [39,43,44].

In this study, a human cell-based screening assay was used to search for compounds capable of inhibiting influenza regulatory protein NS1 based on the modulation of an IFN-β luciferase reporter gene assay.

Potential NS1 inhibitors, derived from a commercial library of 84 structurally diverse molecules [38], were screened for their ability to reverse IFN inhibition by NS1 (Figure 2). The diverse library was selected to screen molecules with different structures/properties to cover a wide drug-like chemical space and activity landscape on different targets [45]. The reliability of the assay was confirmed by employing the known NS1 inhibitor **JJ3297** at the effective concentration of 5 μM.

Two compounds (cpds), **157** and **164,** were identified and confirmed as active in antagonizing NS1 activity (Figure 2 and Figure 3A) without showing non-specific activities on the reporter construct alone or transfected together with the “IFN-inducer” (Figure 3B). The same compounds inhibited A/PR/8/34(H1N1) influenza A virus replication in A549 human lung carcinoma cells, restoring virus-induced IFN-β and ISGs expression (Figure 4 and Figure 5).

**Figure 6:**
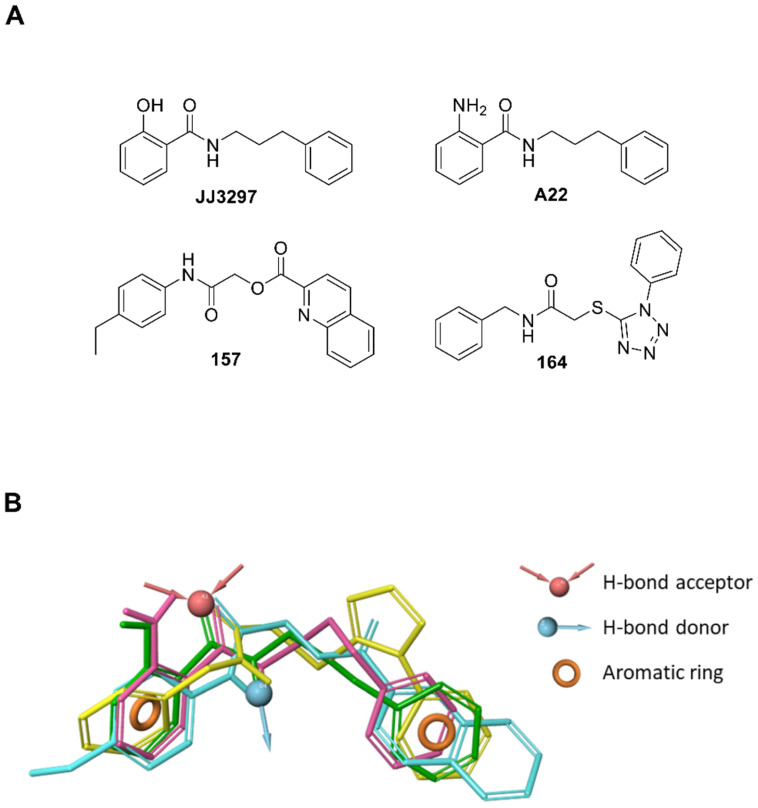
Similarities in the chemical functionalities of compounds **JJ3297**, **A22**, **157,** and **164**. (**A**) 2D structure of the active compounds. (**B**) The pharmacophore model was obtained by aligning all four compounds. The pharmacophore model obtained by aligning the four compounds in the 3D space accounts for two aromatic portions (orange rings), the amide NH as H-bond donor (cyan arrow) and the amide CO as H-bond acceptor (red arrows). The four compounds are represented as green (**JJ3297**), purple (**A22**), cyan (**157**), and yellow (**164**) sticks.

The luciferase assay was sensitive to the cytotoxicity of some inhibitors, and compounds with non-specific toxicity did not support the rebound of the IFN-β driven luciferase signal. In this respect, both **157** and **164** showed an acceptable CC_50_ profile, with **157** possessing a >2-fold higher therapeutic index compared to **164** in a lung epithelial cell line (Figure 4B) [46]. The **JJ3297** NS1 inhibitor [39] is an analog of compound **NSC125044,** previously identified from a chemical library screen as an inhibitor of the slow-growth phenotype of budding yeast expressing NS1 protein [43]. The anti-NS1 activity of **JJ3297** requires a functional RNase L and is not related to direct inhibition of NS1 binding to dsRNA [39]. Subsequently, medicinal chemistry efforts led to the more potent derivative **A22** [47]. It was reported that **A22** had a superior EC_50_ of about 50 nM [44], compared to the 0.8 μM EC_50_ of **JJ3297** [39] and the 8 μM EC_50_ of NSC125044 [43]. Interestingly, evaluation of the structural motif of compounds **157** and **164** revealed a close similarity with **JJ3297** and **A22**, with the four small molecules sharing two aromatic portions spaced by a five-member linker containing an amide function (Figure 6A). To gain better insight into the common chemical functionalities, we developed a 3D ligand-based pharmacophore model using the four compounds as input. A reasonable superimposition between **JJ3297**, **A22**, **157**, and **164** was obtained, with the generated model (Figure 6B) characterized by four features—two aromatic rings and one donor and acceptor group corresponding to the amide moiety.

This observation suggests that the antiviral agents **JJ3297**, **A22**, **157**, and **164** may act via similar mechanisms of action since structurally distinct compounds with comparable pharmacophore patterns are likely to bind the same macromolecule target [48] (i.e., NS1 protein). Furthermore, the proposed 3D model could be exploited in virtual screening campaigns [48,49] to support drug discovery efforts aimed at the rational identification of novel anti-influenza drugs potentially targeting NS1.

It should be mentioned that molecular docking studies and NMR analysis suggested that the mechanism of action of **JJ3297** and **A22** was related to their binding to a hydrophobic pocket within the ED; this region of NS1 is recognized to bind CPSF30, a host factor responsible for the polyadenylation of cellular mRNA [50]. However, the influenza A/PR/8/34(H1N1) virus expresses an NS1 protein bearing two mutations (F103L and M107I) in the ED region that interfere with the binding of CPSF30 and prevent host mRNA processing and subsequent antiviral protein accumulation [51]. Moreover, R108, E125, and G189 residues within the ED of NS1 also interfere with CSF30 binding [52]. Therefore, it is feasible to assume that the mechanism of action of bioactive compounds identified here does not inhibit the NS1-CPSF30 interaction. Rather, **157** and **164** may affect a distinct NS1-host cell factor/s interaction.

Seasonal influenza, as well as the threat of pandemic outbreaks, represent an ongoing global health concern. Previous pandemic outbreaks affected a large proportion of the human population with different degrees of severity, ranging from the devastating H1N1 “Spanish influenza” of 1918/19 to the relatively mild A(H1N1)pdm 09, the “swine flu” of 2009 [53]. Although less dramatic, the cumulative effects of seasonal epidemics that occur in inter-pandemic periods parallel those of pandemics [53]. Avian influenza outbreaks among humans also represent a continuous hazard with a high mortality rate, as in the case of the highly pathogenic H5N1 viruses [4], as well as the H7N9 subtype [5]. Although vaccination represents the best anti-influenza strategy, vaccine production and availability at the beginning of a pandemic may be limited, thus arguing for new anti-influenza drugs, especially those interfering with the virus-cellular signaling machinery [54,55].

## 4. Materials and Methods

### 4.1. Cell Cultures

Human Embryonic Kidney (HEK) 293 cell line and human A549 lung carcinoma cells, obtained from the American Type Culture Collection (ATCC), were grown in Dulbecco’s modified Eagle’s medium (DMEM), (Bio-Whittaker, Cambrex Bio Science, Verviers, Belgium), supplemented with 10% fetal bovine serum (FBS), (Biological Industries, Kibbutz Beit Haemek, Israel), 1mM glutamine and antibiotics (penicillin 100 U/mL, streptomycin 100 µg/mL). All cells were maintained at 37 °C in a 5% CO_2_ atmosphere.

### 4.2. Plasmid Preparation and Purification

Plasmid pBS-IFNβ-Luc (a gift of Dr. Giridhar R. Akkaraju, Department of Biology, Texas Christian University, Fort Worth, TX, USA) consists of the Luciferase gene located downstream of the IFN-β promoter; pCMV2-FLAG-NS1 is an expression vector for FLAG-NS1 fusion proteins; pCMV2-FLAG (Sigma-Aldrich, St. Louis, MO, USA) is the empty vector.

The Immune-stimulatory/ IFN inducer plasmid expression cassette was obtained by de novo gene synthesis (GenScript USA Inc. Piscataway, NJ, USA) by inserting the human Pol III 7SK promoter in the pUC57 vector, followed by a 35 bp sense sequence containing three different nucleotide stretches known to behave as strong type I IFN inducers [56,57,58], followed by a hairpin, a 35 bp antisense, and Pol III terminator sequences, to produce a non-targeting (nt) short hairpin (sh)RNA upon transfection.

The nucleotide blast of the IFN-inducer sequence through the human genomic plus transcript database did not retrieve any match (https://blast.ncbi.nlm.nih.gov/Blast.cgi?PROGRAM=blastn&PAGE_TYPE=BlastSearch&LINK_LOC=blasthome (accessed on 14 June 2023).

IFN-inducer sequence: 5′GTCCTTCAACGGTATGCTGAATTGCAAACCTGTGTTTCAAGAGACACAGGTTTGCAATTCAGCATACCGTTGAAGGACTTTTT3′.

The NS1 nucleotide sequences belong to the A(H1N1)pdm09 influenza virus, isolated in Italy in May 2009, and the corresponding protein sequence is identical to Influenza A virus [(A/District of Columbia/INS229/2009(H1N1))], sequence ID: ADK32781.1. The NS1 cDNA was subsequently generated by de novo gene synthesis (GenScript USA Inc. Piscataway, NJ, USA).

The pBS IFN-β promoter Luciferase and pFlag CMV-2 Flu, NS1 H1N1 Pdm09 plasmids, express proteins upon transfection (firefly luciferase and Flag-NS1, respectively). The expression of luciferase was checked by the luciferase assay, while Flag-NS1 expression was determined by Western blotting. The pUC57 7SK p. IFN inducer plasmid expresses a double-stranded RNA designed to be unable to target any human gene. All plasmids were rigorously prepared using endotoxin-free procedures to avoid unrelated induction of the endogenous type I interferon production.

### 4.3. Transient Transfection, Compound Treatments, and Reporter Gene Assay

Evaluation of the anti-IFN-I activity of NS1 proteins. HEK 293 cells were seeded at 1 × 10^4^ cells/well (100 μL/well) the day before transfection. After 24 h, cells were transiently transfected at 90% confluency in 96 well plates with 25 ng of the IFN-β promoter luciferase reporter construct and different amounts of both IFN-inducer and pFLAG NS1 CMV2 expression vectors, the latter encoding FLAG-tagged NS1 protein belonging to influenza A virus subtypes A(H1N1)pdm09.

Screening of a diverse library of compounds in the IFN-β luciferase system. HEK 293 cells, seeded as described above, were transiently transfected at a 90% confluency in 96 well plates with 25 ng of the IFN-β promoter luciferase reporter construct, 100 ng of the IFN-inducer plasmid, and with 150 ng of the pFLAG CMV2 A(H1N1)pdm09 NS1 construct. A dose response was not performed, considering the small dynamic range of the assay.

Confirmation experiments in the IFN-β luciferase system. HEK 293 cells seeded at 1 × 10^5^/well (1 mL/well) were transiently transfected at a 90% confluency in 12 well plates with 150 ng of the IFN-β promoter luciferase reporter construct, 600 ng of the IFN-inducer plasmid, and with 900 ng of the pFLAG CMV2 A(H1N1)pdm09 NS1 construct.

Equal amounts of plasmid DNA were transfected in each experimental point adding the pFLAG CMV2 empty vector. All DNA plasmid preparations were endotoxin-free by using the EndoFree plasmid Maxi Kit (Qiagen GmbH, Hilden, Germany) according to manufacturer instructions.

The jetPEI (Polyplus-transfection SA, Illkirch FRANCE) reagent was used to transfect HEK 293 cells with the above-mentioned plasmids according to manufacturer instructions. Six hours post-transfection, the transfection medium was removed, replaced with a fresh medium, and cells were treated with compounds for an overall time of forty-two hours.

The ONE Glow Luciferase (glow type) or the Luciferase Assay (flash type) System reagents (Promega) were used to test extracts for luciferase activity 48 h after transfection in a Lumat LB9501 luminometer (E&G Berthold, Bad Wildbad, Germany) according to manufacturer instructions. The same total amount of dimethyl sulfoxide (DMSO) was used at each experimental point. Luciferase activity, measured as relative luciferase units (RLU), was then expressed as a fold of (transcriptional) activation compared to controls (transfected with the reporter plasmids only). The evaluation of the anti-IFN-I activity of different NS1 proteins was conducted using the ONE Glow Luciferase assay. The screening of the diverse library (Figure 2) was also performed using the ONE Glow Luciferase assay and as a single experiment. Active compounds were tested again (Figure 3) using the Luciferase Assay System reagents to eliminate false positive results.

### 4.4. Diverse Library and Other Chemicals Used

The diversity-oriented small molecule selection has already been described [38]. A collection of 950,000 commercially available compounds has been submitted to clustering after binary fingerprints calculation; the most representative molecule from each cluster was chosen, resulting in a library of unique compounds, ideally representative of the starting library chemical space. Briefly, this cheminformatics procedure exploits the conversion of a 2D chemical structure in its fingerprints, a mono-dimensional notation that accounts for compound structural features [59]. Compound fingerprints are then used to compare, and group compounds based on the similarity index (Tanimoto) calculated between all compounds. Therefore, compounds are clustered on the basis of structural similarity. A drug-like subset of 84 small molecules was obtained. All calculations have been carried out using Canvas [60]. The list of compounds composing the diverse library is reported in Table S1 of the Supplementary Materials. The library starts with compound 153 and ends with compound 236. This is just due to an internal progressive nomenclature. The synthesis of **JJ3297** was performed at the Department of Pharmaceutical Sciences, University of Perugia (Italy), according to the literature [47]. Analytical data (1H-NMR and LC-MS) are consistent with that reported in the literature. HPLC purity >95%.

### 4.5. Influenza a Virus Production, Cell Infection, and Viral Titration

Influenza A/PR/8/34(H1N1) virus was grown in the allantoic cavities of 10-day-old embryonated chicken eggs. The allantoic fluid was harvested 48 h after infection and centrifuged at 5000 rpm for 30 min to remove cellular debris. Virus titers were determined by a standard plaque assay [61].

A549 cells were challenged 24 h after plating with influenza A virus at a multiplicity of infection (MOI) of 0.1 as previously described [62]. Mock infection was performed with the same dilution of allantoic fluid from uninfected eggs. Briefly, infected and mock-infected cells were incubated for 1 h at 37 °C and then maintained with fresh medium supplemented with 2% FBS. Tested compounds, dissolved in DMSO, were diluted in DMEM to a final concentration of 50 µM and then added to A549 cell monolayer 3 h before infection. The highest DMSO concentration present in the culture medium was 0.05%. The same DMSO concentration was added to control cells. Supernatants from infected cells were recovered 24 h post-infection (p.i) to measure viral titer by hemagglutinating activity [63]. The Inhibitory Concentration to reduce virus yield by 50% (IC_50_) was calculated by plotting HAU values obtained from A549-infected cells, derived from three separate experiments, treated with different concentrations of compounds (cpds) compared to those from untreated cells (considered as 100%). A linear regression analysis was used to calculate the IC_50_ concentration.

### 4.6. Neutral Red Uptake Assay

Cytotoxic concentration to cause death to 50% of viable cells (CC_50_) was calculated by neutral red uptake assay (NRU) assay [64]. In brief, 1 × 10^4^ A549 cells were seeded in 96-well plates and exposed to different concentrations of cpds **157**, **164** (25, 50, 100, 200, and 400 μM) or equivalent volumes of DMSO as a control for 48 h. At the end of the exposure time, cells were washed with phosphate-buffered saline (PBS) before being incubated for 3 h in a medium supplemented with neutral red (50 μg/mL). The medium was washed off rapidly with PBS, and cells were incubated for a further 15′ at R.T. in a mixture of 1% acetic acid and 50% ethanol to extract the dye. Absorbance was then measured at 540 nm using a micro-plate reader (Biorad). Neutral red powder was purchased from Sigma-Aldrich. The percentage of cell death for each compound concentration, compared to the corresponding DMSO amount in untreated controls (*y*-axis), was plotted against increasing cpd concentrations (*x*-axis). Data were transformed in log scale [*x* = log(*x*)], and a linear regression analysis was used to calculate the CC_50_ concentration.

### 4.7. Quantitative Real-Time Reverse Transcription-PCR (qRT-PCR)

Total RNA was extracted from infected and/or treated A549 cells using the RNeasy total RNA extraction kit (Qiagen, Hilden, Germany). Total RNA was subjected to DNase treatment with RNase-free DNase (Qiagen) and then reverse transcribed with a High-Capacity cDNA reverse transcription kit (Applied Biosystems, Warrington, UK) according to the manufacturer’s instructions. cDNA was subjected to quantitative real-time PCR on ABI 7000 sequence detection system (Applied Biosystems) by using SYBR green PCR master mix (Applied Biosystems). Primers used for Quantitative Real-Time Reverse Transcription-PCR (qRT-PCR) were as follows:

IFN-β forward primer 5′-GCAGCAGTTCCAGAAGGAG-3′ and reverse primer 5′-GCCAGGAGGTTCTCAACAAT-3′, RNAseL forward primer 5′-GAAGCCGCTGTGTATGGTAA-3′ and reverse primer 5′-CGCTCTTGATCCTCCTTTGT-3′, PKR forward primer 5′-AAACAATTGGCCGCTAAACT-3′ and reverse primer 5′-ATTCAGAAGCGAGTGTGCTG-3′, and OAS forward primer 5′-GGTGGTAAAGGGTGGCTCC-3′ and reverse primer 5′-ACAACCAGGTCAGCGTCAGAT-3′.

Transcript levels were normalized to glyceraldehyde-3-phosphate dehydrogenase (GAPDH) (forward, 5′-GGGTGTGAACCATGAGAAG-3′; reverse, 5′-GCTAAGCAGTTGGTGGTGC-3′) as an internal control and expressed as fold of increase according to the Δ*C_T_* methods (means ± standard deviations). In the case of qRT-PCR performed using mRNA derived from infected or infected and treated A549 cells, a further normalization was performed for the level of viral replication using Hemagglutination data.

### 4.8. Pharmacophore Modeling

Compounds **JJ3297**, **A22**, **157**, and **164** were built using the fragment library tool of Maestro GUI [65] and used as a training set for the development of a common feature pharmacophore model. MacroModel [66] was used to conduct a conformational search on these compounds. The maximum number of steps per molecule was set to 10,000 in order to improve conformational sampling. The Polak–Ribiere conjugate gradient method was used to minimize conformers, with a maximum of 500 minimization steps and 0.0005 kJ/(Å mol) as the gradient convergence threshold. The explored energy window for the creation of the molecule conformations was 10.04 kcal/mol. The created conformations were used in the development of a common feature pharmacophore model by using Phase [67,68]. Pharmacophore models in which at least one training set compound showed a low fitness value (i.e., <1.8) were discarded. The best-identified hypothesis (ADRR) was characterized by the following scores: PhaseHypoScore = 1.089; BEDROC score = 0.852; Survival score = 3.966. The fitness values for the training set compounds were: **JJ3297** = 3.0; **A22** = 2.36; **157** = 2.12; **164** = 2.00.

### 4.9. Statistical Analysis

The GraphPad Prism software for Windows v5.0 (Dotmatics, Bishop’s Stortford, UK) was used for the statistical analysis through the “two tailed paired/unpaired t test” or, in the case of multiple comparisons, using the “one-way analysis of variance” (ANOVA), followed by the Newman–Keuls post hoc test. Values of at least *p* < 0.05 (*), *p* < 0.01 (**), and *p* < 0.001 (***) were considered to be statistically significant.

## 5. Conclusions

This study demonstrates the identification of small molecules that inhibit the replication of influenza using a simplified early drug discovery process. The essential features of the cell-based assay involve the selection of an appropriate molecular target (in this case, the multifunctional viral NS1 protein), an optimized cellular gene expression assay (e.g., IFN-β promoter luciferase reporter and IFN-inducer plasmid), and a structurally diverse small molecule library. The choice of using a “transient” assay permits a concentration-dependent flexibility of the conditions of the assay and the compounds to be evaluated. Nevertheless, this approach is a starting point in the development of stable cellular platforms expressing the IFN-β promoter and the “IFN-inducer” shRNA for high throughput screening campaigns. Moreover, the flexible cellular assay can also be adapted to search for inhibitors of relevant viral targets belonging to other pathogenic viruses.

## Data Availability

The data presented in this study are available within the article or supplementary material.

## Supplementary Materials

The following supporting information can be downloaded at https://www.mdpi.com/article/10.3390/ijms241310495/s1.
