# Supplementary material for: Identification of Anti-Influenza A Compounds Inhibiting the Viral Non-Structural Protein 1 (NS1) Using a Type I Interferon-Driven Screening Strategy
Source: Int J Mol Sci. 2023 Jun 22;24(13):10495. doi: 10.3390/ijms241310495 (PMC10341698; doi:10.3390/ijms241310495)
Supplement: Supplementary file 1 [file ijms-24-10495-s001.zip › Figure S1.pdf]

compound 157

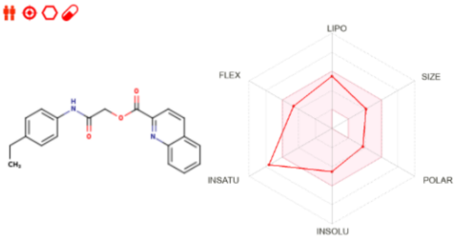

SMILES CCc1ccc(cc1)NC(=O)COC(=O)c1ccc2c(n1)cccc2

Physicochemical Properties	
Formula	C20H18N2O3
Molecular weight	334.37 g/mol
Num. heavy atoms	25
Num. arom. heavy atoms	16
Fraction Csp3	0.15
Num. rotatable bonds	7
Num. H-bond acceptors	4
Num. H-bond donors	1
Molar Refractivity	96.63
TPSA	68.29 Å²

Lipophilicity	
Log $P_{o/w}$ (iLOGP)	2.87
Log $P_{o/w}$ (XLOGP3)	4.08
Log $P_{o/w}$ (WLOGP)	3.40
Log $P_{o/w}$ (MLOGP)	2.59
Log $P_{o/w}$ (SILICOS-IT)	3.81
Consensus Log $P_{o/w}$	3.35

Water Solubility	
Log *S* (ESOL)	-4.50
Solubility	1.07e-02 mg/ml ; 3.20e-05 mol/l
Class	Moderately soluble
Log *S* (Ali)	-5.22
Solubility	2.02e-03 mg/ml ; 6.04e-06 mol/l
Class	Moderately soluble
Log *S* (SILICOS-IT)	-7.07
Solubility	2.82e-05 mg/ml ; 8.43e-08 mol/l
Class	Poorly soluble

Pharmacokinetics	
GI absorption	High
BBB permeant	Yes
P-gp substrate	No
CYP1A2 inhibitor	Yes
CYP2C19 inhibitor	Yes
CYP2C9 inhibitor	Yes
CYP2D6 inhibitor	Yes
CYP3A4 inhibitor	Yes
Log K_p (skin permeation)	-5.44 cm/s

Druglikeness	
Lipinski	Yes; 0 violation
Ghose	Yes
Veber	Yes
Egan	Yes
Muegge	Yes
Bioavailability Score	0.55

Medicinal Chemistry	
PAINS	0 alert
Brenk	0 alert
Leadlikeness	No; 1 violation: XLOGP3>3.5
Synthetic accessibility	2.43

compound 164

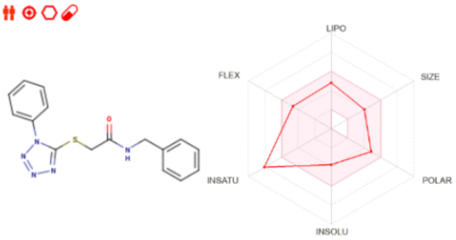

SMILES O=C(CSc1nnnn1c1ccccc1)NCc1ccccc1

Physicochemical Properties	
Formula	C16H15N5OS
Molecular weight	325.39 g/mol
Num. heavy atoms	23
Num. arom. heavy atoms	17
Fraction Csp3	0.12
Num. rotatable bonds	7
Num. H-bond acceptors	4
Num. H-bond donors	1
Molar Refractivity	87.98
TPSA	98.00 Å²

Lipophilicity	
Log $P_{o/w}$ (iLOGP)	2.95
Log $P_{o/w}$ (XLOGP3)	2.91
Log $P_{o/w}$ (WLOGP)	1.92
Log $P_{o/w}$ (MLOGP)	2.67
Log $P_{o/w}$ (SILICOS-IT)	1.84
Consensus Log $P_{o/w}$	2.46

Water Solubility	
Log *S* (ESOL)	-3.78
Solubility	5.45e-02 mg/ml ; 1.68e-04 mol/l
Class	Soluble
Log *S* (Ali)	-4.63
Solubility	7.65e-03 mg/ml ; 2.35e-05 mol/l
Class	Moderately soluble
Log *S* (SILICOS-IT)	-5.60
Solubility	8.25e-04 mg/ml ; 2.54e-06 mol/l
Class	Moderately soluble

Pharmacokinetics	
GI absorption	High
BBB permeant	No
P-gp substrate	No
CYP1A2 inhibitor	Yes
CYP2C19 inhibitor	Yes
CYP2C9 inhibitor	Yes
CYP2D6 inhibitor	No
CYP3A4 inhibitor	No
Log K_p (skin permeation)	-6.22 cm/s

Druglikeness	
Lipinski	Yes; 0 violation
Ghose	Yes
Veber	Yes
Egan	Yes
Muegge	Yes
Bioavailability Score	0.55

Medicinal Chemistry	
PAINS	0 alert
Brenk	0 alert
Leadlikeness	Yes
Synthetic accessibility	2.55

Figure S1. Prediction of ADME profile and physicochemical and pharmacokinetic properties of compounds **157** and **164**.
