# Supplementary material for: Identification of Anti-Influenza A Compounds Inhibiting the Viral Non-Structural Protein 1 (NS1) Using a Type I Interferon-Driven Screening Strategy
Source: Int J Mol Sci. 2023 Jun 22;24(13):10495. doi: 10.3390/ijms241310495 (PMC10341698; doi:10.3390/ijms241310495)
Supplement: Supplementary file 1 [file ijms-24-10495-s001.zip › Table S1.pdf]

Supporting information

Table S1. ID, *supplier code* and structure of the 84 screened compounds composing the diverse library.

**153**<br>*T0340-1456*<br>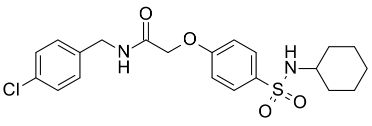	**154**<br>*T0306-9137*<br>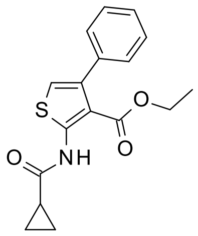	**155**<br>*T0505-5794*<br>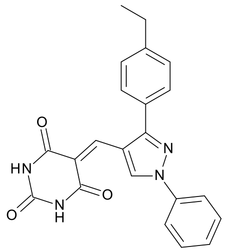
**156**<br>*T0366-3184*<br>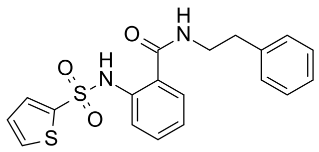	**157**<br>*T0506-5281*<br>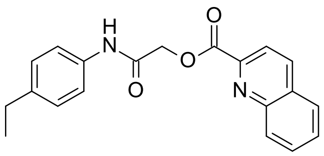	**158**<br>*T0511-0248*<br>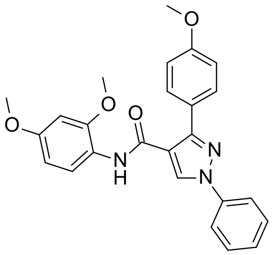
**159**<br>*T0511-8649*<br>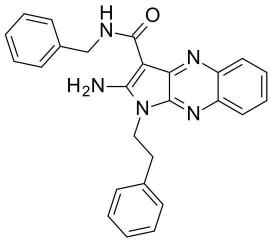	**160**<br>*T0515-0254*<br>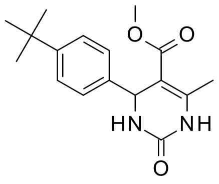	**161**<br>*T0518-2424*<br>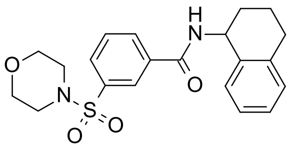

**162**<br>*T0316-6988*<br>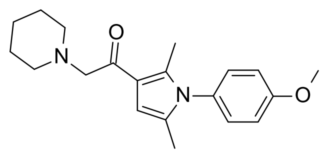	**163**<br>*T0520-9292*<br>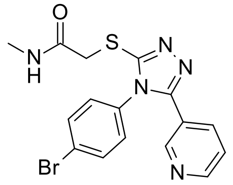	**164**<br>T0301-3213<br>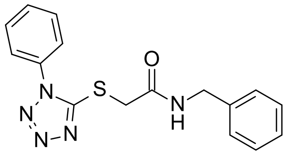
**165**<br>*T5263448*<br>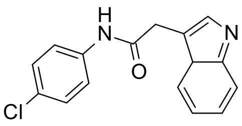	**166**<br>*T5272509*<br>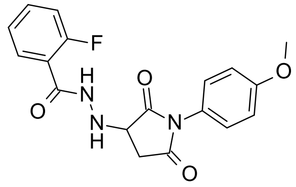	**167**<br>*T5284258*<br>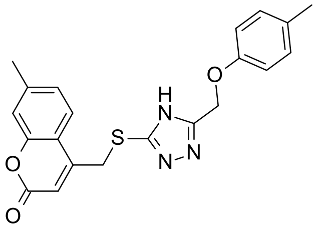
**168**<br>*T5305571*<br>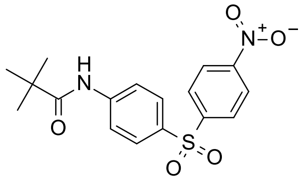	**169**<br>*T5308219*<br>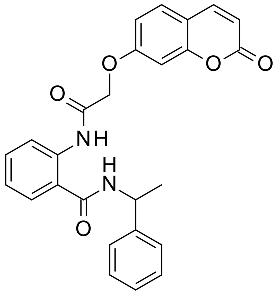	**170**<br>*T5316287*<br>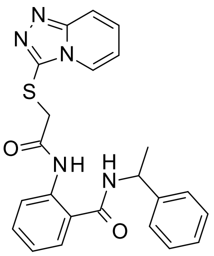
**171**<br>*T5316819*<br>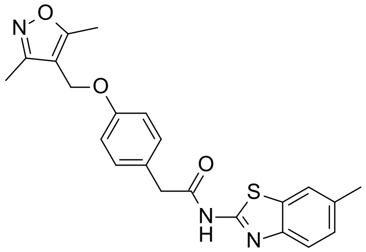	**172**<br>*T5318448*<br>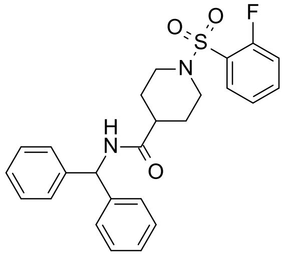	**173**<br>*T5329791*<br>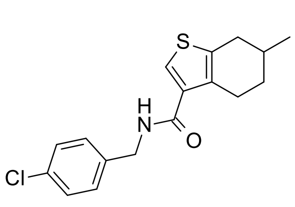

**174**<br>*T5330349*<br>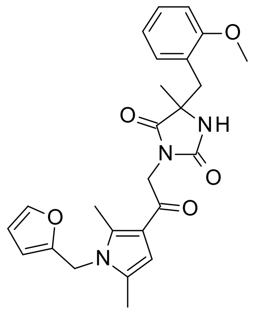	**175**<br>*T5335333*<br>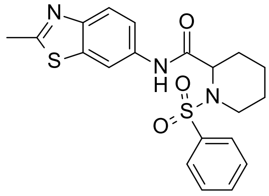	**176**<br>*T5393793*<br>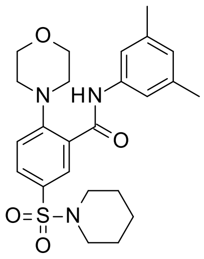
**177**<br>*T5396052*<br>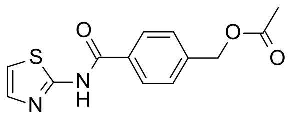	**178**<br>*T5450172*<br>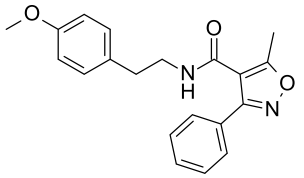	**179**<br>*T5460509*<br>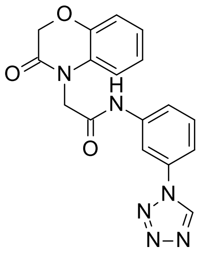
**180**<br>*T5460611*<br>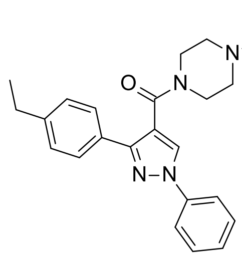	**181**<br>*T0358-2870*<br>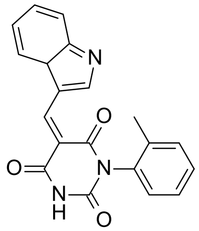	**182**<br>*T5465380*<br>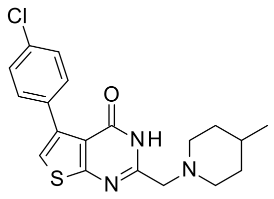
**183**<br>*T5558390*<br>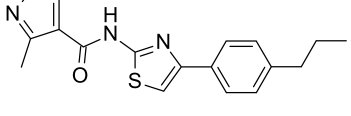	**184**<br>*T5377312*<br>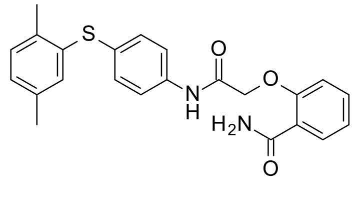	**185**<br>*T5570620*<br>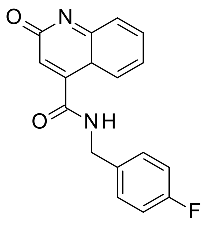

**186**<br>*T5571734*<br>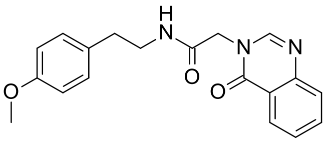	**187**<br>*T5594464*<br>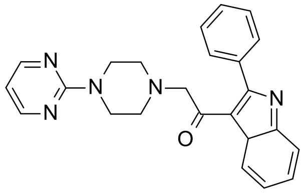	**188**<br>*T5603735*<br>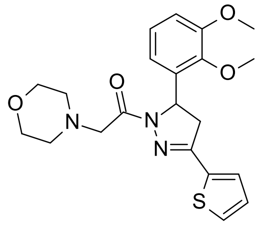
**189**<br>*T1329-3854*<br>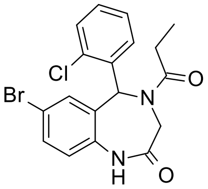	**190**<br>*T5635981*<br>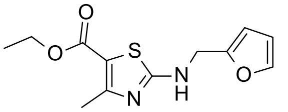	**191**<br>*T5622820*<br>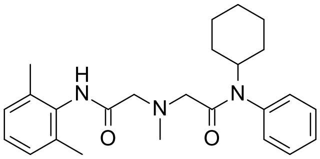
**192**<br>*T5661504*<br>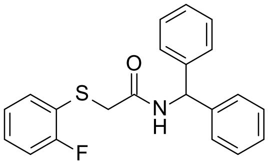	**193**<br>*T5659078*<br>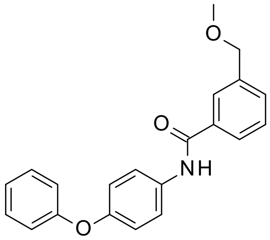	**194**<br>*T5687563*<br>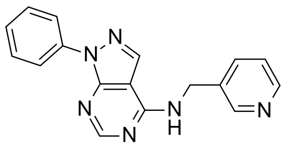
**195**<br>*T5719429*<br>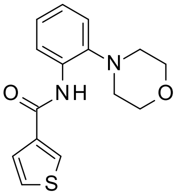	**196**<br>*T5722483*<br>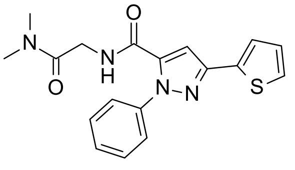	**197**<br>*T1305-9117*<br>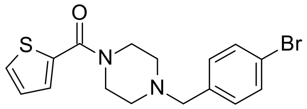

**198**<br>*T5724349*<br>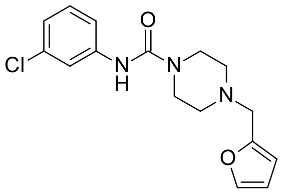	**199**<br>*T0391-8750*<br>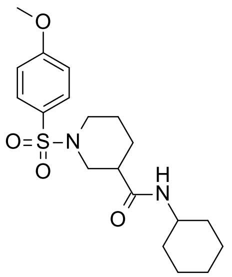	**200**<br>*T0376-3307*<br>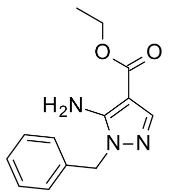
**201**<br>*T5804390*<br>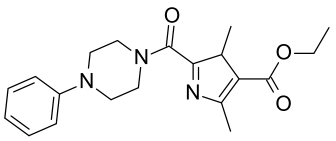	**202**<br>*T5879448*<br>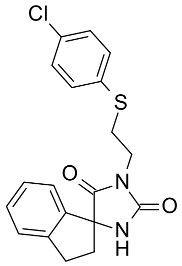	**203**<br>*T5883275*<br>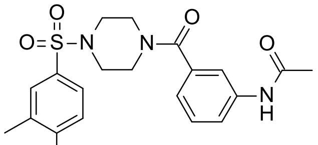
**204**<br>*T5928450*<br>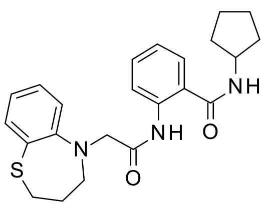	**205**<br>*T5956270*<br>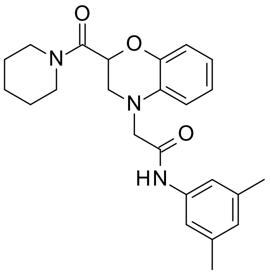	**206**<br>*T0335-3920*<br>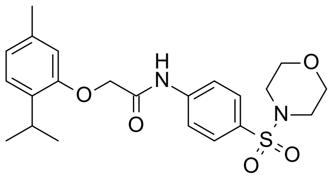
**207**<br>*T6075418*<br>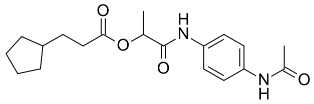	**208**<br>*T6111920*<br>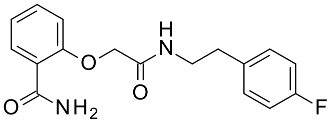	**209**<br>*T6117467*<br>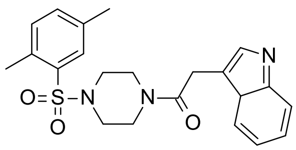

**210**<br>*T6246601*<br>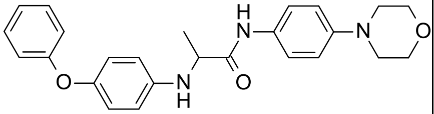	**211**<br>*T6285788*<br>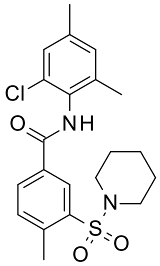	**212**<br>*T6304972*<br>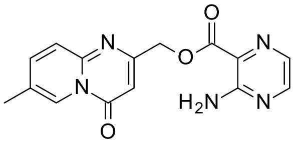
**213**<br>*T6310799*<br>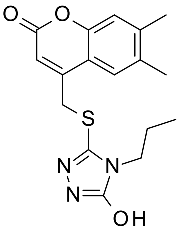	**214**<br>*T6317162*<br>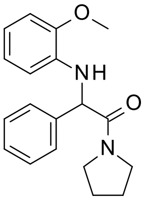	**215**<br>*T6327498*<br>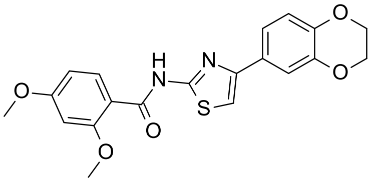
**216**<br>*T6421291*<br>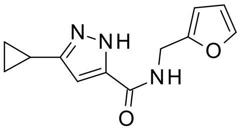	**217**<br>*T0319-8439*<br>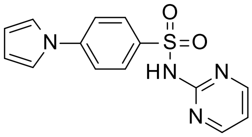	**218**<br>*T5985309*<br>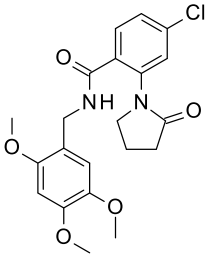
**219**<br>*T6568785*<br>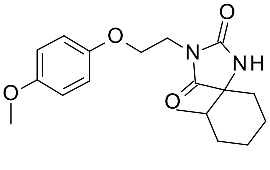	**220**<br>*T6586890*<br>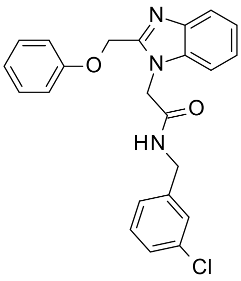	**221**<br>*T5890666*<br>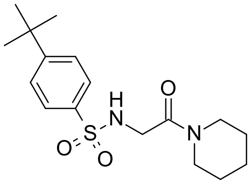

**222**<br>*T5889788*<br>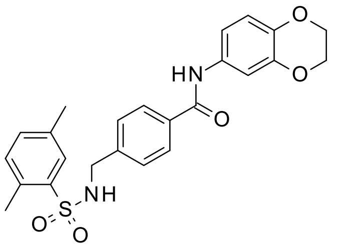	**223**<br>*T5389018*<br>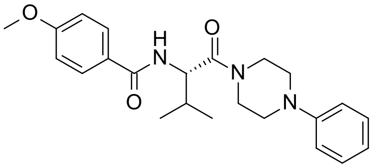	**224**<br>*T5437635*<br>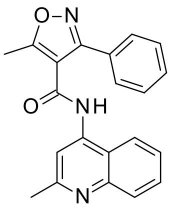
**225**<br>*T0508-2915*<br>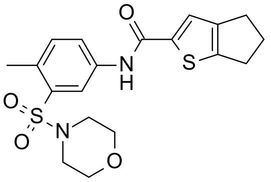	**226**<br>*T0518-5559*<br>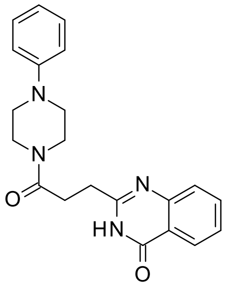	**227**<br>*T5264829*<br>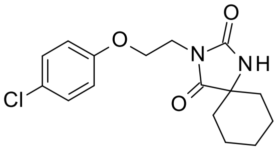
**228**<br>*T1323-1121*<br>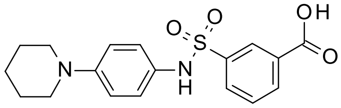	**229**<br>*T5540131*<br>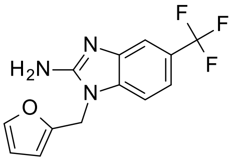	**230**<br>*T5562138*<br>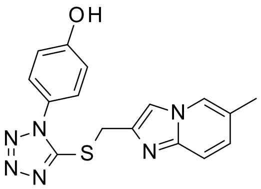
**231**<br>*T5291479*<br>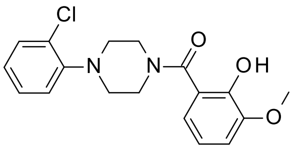	**232**<br>*T6506923*<br>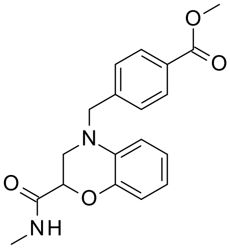	**233**<br>*T6602344*<br>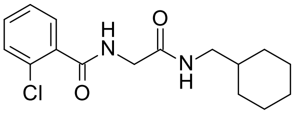

**234**<br>*T0322-9346*<br>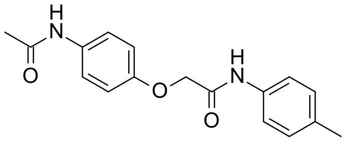	**235**<br>*T5288693*<br>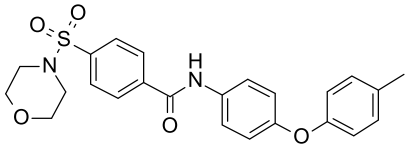	**236**<br>*T0327-2587*<br>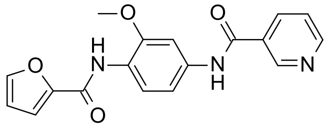
